# Influence of heart rate control on exercise capacity and quality of life in patients with permanent atrial fibrillation

**DOI:** 10.1186/s12872-019-01293-3

**Published:** 2019-12-21

**Authors:** Fei She, Yuan Ma, Yi Li, Lei Li, Weixian Xu, Hongyan Wang, Ming Cui

**Affiliations:** 1 Department of Cardiology, Beijing Tsinghua Changgung Hospital, School of Clinical Medicine, Tsinghua University, Beijing, China; 2 Department of Cardiology, Peking University Third Hospital, 49 North garden Road, Haidian District, Beijing, China; 3 Key Laboratory of Cardiovascular Molecular Biology and Regulatory Peptides, Ministry of Health, Beijing, China; 4 Key Laboratory of Molecular Cardiovascular Sciences, Ministry of Education, Beijing, China

**Keywords:** Heart rate control, Permanent atrial fibrillation, Peak oxygen uptake, Peak metabolic equivalent, Anaerobic threshold, Quality of life

## Abstract

**Background:**

The optimal level of heart rate (HR) control in patients with atrial fibrillation (AF) is unknown. To assess the effect of rate control on cardiopulmonary exercise capacity and quality of life (QoL) in permanent AF.

**Methods:**

One hundred forty-three patients with permanent AF were enrolled in this study. All patients received rate control medications and were followed up for 1 year. After 1-year therapy, the exercise capacity and QoL were evaluated by cardiopulmonary exercise testing (CPET) and 36-item Short-Form Health Survey, respectively. Data were compared by dividing the patients according to the following criteria: (1) whether the resting HR was ≤80 or > 80 bpm; (2) whether the exercise HR during moderate exercises on CPET was ≤110 or > 110 bpm; and (3) whether the resting HR was ≤80 bpm and exercise HR was ≤110 bpm.

**Results:**

No significant differences in peak oxygen uptake, peak metabolic equivalent, and anaerobic threshold were found between the strict control and lenient control groups. Both physical component summary (PCS) and mental component summary (MCS) were significantly higher for the strict rate control group than for the lenient control group. The single-factor correlation analysis revealed a negative correlation between resting HR and both PCS and MCS. The multivariable linear regression analysis indicated that both exercise HR and duration of AF linearly correlated with PCS and MCS.

**Conclusions:**

Therefore, in patients with permanent AF, exercise capacity may not be affected by the stringency of rate control, and strict rate control may be associated with better QoL.

## Background

The increasing prevalence of atrial fibrillation (AF) poses heavy public health burden in terms of high cardiovascular and all-cause mortality [1].

Despite an improved outcome due to advanced ablation techniques [1–4], the recurrence of AF is common and a significant number of patients continue to experience complications [5].

AF is associated with stroke and heart failure [3, 6]. Previous studies established that the rate control strategy was not inferior to rhythm control for major clinical events in patients with AF [7–10]. Therefore, rate control has become the front-line therapy in managing AF. The optimal level of heart rate (HR) control, however, remains unknown. Recently, the Rate Control Efficacy in Permanent Atrial Fibrillation II (RACE II) trial revealed that lenient rate control was as effective as strict rate control with respect to morbidity and mortality in patients with permanent AF [1]. However, the evaluation of the quality of life (QoL) in RACE II trial was based only on the rehospitalization rates. Meanwhile, data concerning the influence of rate control on cardiopulmonary exercise capacity in permanent AF are insufficient. Therefore, the aim of this study was to assess the effect of rate control on cardiopulmonary exercise capacity and QoL in permanent AF.

## Methods

### Patient population

A total of 143 patients (100 men, mean age 67.0 ± 9.0 years) from Peking University Third Hospital between September 2010 and May 2011 were examined (Table 1). All patients suffered from permanent AF of New York Heart Association (NYHA) functional classes I–II. The permanent AF is defined as Continuous AF lasting > 1 year and accepted by the patient and physician [2]. Informed consent regarding the cardiopulmonary exercise test was obtained from all patients.

**Table 1:** Baseline characteristics of the study patients

Characteristic	
Total population (*n*)	143
Age (year)	67.0 ± 9.0
Male (*n*, %)	100 (70.0)
Smoke (*n*, %)	27 (18.9)
Duration of AF (month)	60 (36–108)
Hypertension (*n*, %)	72 (50.3)
Coronary heart disease (*n*, %)	21 (14.7)
Valvular disease (*n*, %)	5 (3.5)
Dilated cardiomyopathy (*n*, %)	0 (0)
Diabetes (*n*, %)	20 (14.0)
BMI (kg/m^2^)	26.4 ± 3.3
Abdominal girth (cm)	93.4 ± 9.5
Blood pressure (mm Hg)	
Systolic	124.7 ± 17.3
Diastolic	77.8 ± 10.8
LVEF (%)	62.4 ± 13.6
NYHA functional class (*n*, %)	
I	97 (67.8)
II	46 (32.2)

*BMI* body mass index, *LVEF* left ventricular ejection fraction, *NYHA* New York Heart Association

The exclusion criteria were as follows: (1) paroxysmal AF; (2) resting HR > 110 bpm; (3) symptomatic heart failure (NYHA III–IV) and previously hospitalized due to heart failure in the last 3 months; (4) acute myocardial infarction, unstable angina, myocarditis, pericarditis, rheumatic fever, infective endocarditis, and postoperative cardiac surgery within 3 months; (5) other severe diseases (stroke, HIV infection, cancer, severe primary liver or kidney disease, and chronic pulmonary diseases including pulmonary embolism); (6) any causes of anemia; (7) thyroid gland dysfunction or thyroid function returning to normal in less than 3 months; and (8) limited mobility [4, 5].

### Study protocol

Data on age, gender, duration of AF, concomitant disease, medical history, biochemical index, and other clinical characteristics were collected. Rate control was instituted with beta-blockers, nondihydropyridine calcium-channel blockers, and digoxin alone or in combination at various doses at the discretion of outpatient physicians (Table 2). Outpatient visits occurred every 4 weeks. After 1-year therapy, each patient underwent a cardiopulmonary exercise test to record resting heart rate (HR), exercise HR, peak oxygen uptake (VO_2_peak), peak metabolic equivalent (MET peak), and anaerobic threshold (VO_2_AT) and also completed the 36-item Short-Form Health Survey (SF-36) quality-of-life questionnaire. Target strict resting HR control was defined as HR ≤80 bpm based on the criteria of Pharmacological Intervention in Atrial Fibrillation study [11]. Target strict exercise HR control was defined as ventricular rate ≤ 110 bpm according to criteria established by the Atrial Fibrillation Follow-Up Investigation of Rhythm Management [12]. Exercise capability and QoL were compared by dividing the patient population according to the following criteria: (1) whether the resting HR was ≤80 or > 80 bpm; (2) whether the exercise HR during moderate exercises on the cardiopulmonary exercise test (CPET) was ≤110 or > 110 bpm; and (3) whether the resting HR was ≤80 bpm and exercise HR was ≤110 bpm [1, 3].

**Table 2:** Primary pharmacological therapy

Medications	*N* (%)
β-Blocker	82 (57.5)
Digoxin	11 (8)
Calcium-channel blocker	13 (9.2)
β-Blocker + calcium-channel-blocker	30 (20.7)
β-Blocker + calcium-channel-blocker + digoxin	7 (4.6)

### Cardiopulmonary exercise test

All patients underwent the CPET on a motorized treadmill (Max-2; Physio-Dyne, USA) according to a maximal symptom-limited, modified Bruce protocol [13]. Ratings of perceived exertion and blood pressure were recorded every 1 min for each stage. In the meantime, oxygen uptake (VO_2_) was assessed every 30 s during the test. A standard 12-lead electrocardiogram was continuously recorded. The resting HR was measured in both groups by means of 12-lead electrocardiography after 15 min of rest in the supine position. The exercise HR was measured during a moderate exercise performed for the onset of 3-min exercise at 2.7 km/h with 5% gradient, which was comparable to the ordinary physical activity.

### Quality-of-life questionnaire

QoL was evaluated using the SF-36. In clinical practice, it is the most commonly used questionnaire to assess QoL and has been validated in several cardiovascular diseases, including AF [14]. The SF-36 is a standardized, validated, health-related quality-of-life survey. It consists of 36 items covering 8 subscales [15, 16]. For each subscale, scores are transformed to a scale ranging from 0 to 100. The physical component summary (PCS) and mental component summary (MCS) are calculated and used for evaluating physical health and mental health, respectively. Higher scores indicated better QoL.

### Statistical analysis

All analyses were performed with the software package SPSS 13.0 (SPSS, Inc., IL, USA). Continuous outcome variables that followed a normal distribution were presented as mean ± standard deviation, and tested using the Student’s *t* test. Variables that did not follow a normal distribution were presented as median (IQR) and tested using the Wilcoxon rank-sum test. Categorical variables were shown as rate or proportion and tested using the Pearson chi-square test. Multivariable logistic regression models were used. A *P* value less than 0.05 was considered statistically significant.

## Results

All patients suffered from permanent AF, the mean age was 67.0 ± 9.0 years old, which consistent with previous studies in China [17]. The data were compared using three grouping methods according to HR. No significant differences in age, duration of AF, concomitant disease, history of smoke, BMI, and abdominal girth were observed. The strict rate control group included more male patients compared with the lenient rate control group. The mean HR of resting and during moderate a exercise was significantly lower among participants from the strict rate control group than among those from the lenient control group. No significant differences were found in the use of β-blockers, calcium-channel blockers, and digoxin. The blood biochemical test revealed no significant differences in the ALT, Cr, UA, T-CHO, and blood glucose levels between the two groups (results not shown).

Regarding the resting HR of 80 bpm as a cutoff point, no significant differences in VO_2_peak/kg, MET peak, and VO_2_AT/kg were found. Both PCS and MCS were significantly higher among participants from the strict rate control group than among those from the lenient control group (*P* < 0.05, Table 3). The single-factor correlation analysis revealed a negative correlation between resting HR and both PCS and MCS. The multivariable linear regression analysis indicated that resting HR did not linearly correlate with PCS or MCS (Additional file 1: Tables S1 and S2).

**Table 3:** Cardiopulmonary exercise capacity and quality of life with respect to resting HR

Variable	Resting HR ≤80 bpm (*n* = 77)	Resting HR > 80 bpm (*n* = 66)	*P*
CPET			
VO_2_peak/kg [mL/(kg·min)]	21.5 ± 5.3	22.1 ± 6.0	0.560
MET peak	6.3 ± 1.6	6.0 ± 1.7	0.860
VO_2_AT/kg [mL/(kg·min)]	20.9 ± 4.9	22.3 ± 6.6	0.261
SF-36 score			
PCS	356.2 ± 37.7	318.2 ± 51.6	0.001
MCS	359.4 ± 40.5	314.9 ± 52.2	0.000

*AT* Anaerobic threshold, *CPET* cardiopulmonary exercise testing, *HR* heart rate, *MCS* mental component summary, *MET peak* peak metabolic equivalent, *PCS* physical component summary, *SF-36* Short-form health survey, *VO*_*2*_*peak* Peak oxygen uptake

Taking the exercise HR of 110 bpm into consideration, no significant differences in VO_2_peak/kg, MET peak, and VO_2_AT/kg were found between the two groups. PCS and MCS were significantly higher in the strict rate control group than in the lenient rate control group (*P* < 0.05, Table 4). Multivariable linear regression analysis indicated that both exercise HR and duration of AF linearly correlated with PCS and MCS (Additional file 1: Tables S3 and S4).

**Table 4:** Cardiopulmonary exercise capacity and quality of life with respect to exercise HR

Variable	Exercise HR ≤110 bpm (*n* = 61)	Exercise HR > 110 bpm (*n* = 82)	*P*
CPET			
VO_2_peak/kg [mL/(kg·min)]	21.8 ± 5.3	21.7 ± 5.9	0.940
MET peak	6.4 ± 1.6	6.2 ± 1.7	0.578
VO_2_AT/kg [mL/(kg·min)]	21.0 ± 5.4	21.6 ± 5.8	0.790
SF-36 score			
PCS	358.0 ± 40.0	320.7 ± 48.2	0.001
MCS	362.0 ± 39.7	317.4 ± 51.2	0.000

*CPET* Cardiopulmonary exercise testing, *HR* Heart rate, *MCS* Mental component summary, *MET peak* Peak metabolic equivalent, *PCS* Physical component summary, *SF-36* short-form health survey, *VO*_*2*_*peak* peak oxygen uptake

According to both resting HR and exercise HR, strict rate control was defined as resting HR ≤80 bpm and HR during moderate exercise ≤110 bpm, whereas lenient rate control was defined as resting HR > 80 bpm or exercise HR > 110 bpm. The CPET showed no significant differences in VO_2_peak/kg, MET peak, and VO_2_AT/kg. Patients in the strict rate control group had higher PCS and MCS scores of SF-36 than patients in the lenient control group (*P* < 0.05, Table 5).

**Table 5:** Cardiopulmonary exercise capacity and quality of life with respect to the combination of resting HR and exercise HR

Variable	Resting HR ≤80 bpm and Exercise HR ≤110 bpm (*n* = 53)	Resting HR > 80 bpm or Exercise HR > 110 bpm (*n* = 90)	*P*
CPET			
VO_2_peak/kg [mL/(kg·min)]	22.3 ± 4.7	21.5 ± 6.0	0.385
METs peak	6.6 ± 1.4	6.0 ± 1.7	0.158
VO_2_AT/kg [mL/(kg·min)]	21.9 ± 4.7	21.2 ± 6.1	0.644
SF-36 score			
PCS	362.1 ± 34.4	323.3 ± 49.8	0.001
MCS	367.2 ± 33.7	320.4 ± 52.1	0.000

*CPET* cardiopulmonary exercise testing, *HR* heart rate, *MCS* mental component summary, *MET peak* peak metabolic equivalent, *PCS* physical component summary, *SF-36* short-form health survey, *VO*_*2*_*peak* peak oxygen uptake

## Discussion

An irregular rhythm and a rapid ventricular rate in AF can lead to symptoms including palpitations, dyspnea, fatigue, and dizziness [3, 6]. Adequate control of ventricular rate may reduce symptoms and improve hemodynamics by allowing enough time for ventricular filling and prevention of tachycardiomyopathy [18]. Therefore, in theory, strict rate control should be able to improve the exercise capacity and QoL.

The results of the present study were in disagreement with previous findings, which reported that lenient rate control was not inferior to strict rate control in terms of QoL in patients with permanent AF [1, 19, 20]. Strict rate control was found to be associated with better QoL. Furthermore, the exercise HR was an independent predictor of QoL. It should be noted that exercise HR was evaluated by a 6-min walk test (6MWT) in previous studies [21]. However, the limitation of 6WMT was that patients determined their own speed, resulting in a difference in work intensity and total distance walk over the test. The modified Bruce protocol was used in CPET to standardize the workload for all patients in each stage of the test. The same workload was established for all patients in each stage of the test. For improving cardiopulmonary capacity in permanent AF, the data suggested that strict rate control had no significant influence.

The next question was why strict rate control did not affect exercise capability in AF. One explanation was that patients with AF enrolled in the present study had no severe heart and respiratory failure. Hence, the impact of ventricular rate with cardiopulmonary exercise capability could be well compensated. In addition, as a cross-sectional design, this study focused only on the correlation between HR and exercise capability.

Additional analysis for the association between each SF-36 items and heart rate demonstrated that items related to symptoms like vitality, general health and social functioning showed higher correlation with heart rate, while physical functioning showed poor correlation with heart rate (Additional file 1: Table S5). This may indicate that strict ventricular rate control might improve QoL but not efficient enough to enhance exercise capacity. There might be several reasons as below: First, strict rate control could alleviate the palpitations and other symptoms related to elevated HR during exercise. Second, exercise could increase oxygen consumption, while a fast and irregular HR was usually associated with an insufficient cardiac output, which could not fulfill the requirement of the body. These situations could be improved by strict rate control. Finally, fatigue caused by exercise could aggravate the arrhythmia-related symptoms. The control of HR could relieve these symptoms. Of note, the exercise HR in this study was defined as the ventricular heart rate with the MET value of 3, which was comparable to the ordinary physical activity. Therefore, the exercise HR was more valuable for evaluating cardiopulmonary exercise capacity and QoL.

The single-factor correlation analysis revealed a negative correlation between resting HR and both PCS and MCS. The multiple regression analysis demonstrated no linear regression relationship between resting HR, PCS, and MCS. These results indicated that resting HR might not be an independent predictor of QoL. On the contrary, as a result of insufficient sample size, the multiple regression analysis showed that resting HR negatively correlated with PCS and MCS; the *P* value was close to 0.05. If the sample size was continuously expanded, this might be a result of a linear relationship. Accordingly, the possibility that the resting HR could be used as an independent predictor of QoL could not be precluded.

### Study limitations

This was a nonrandomized study. However, the data were prospectively collected from the patients. Another limitation was related to the diversity of drugs prescribed by various physicians to achieve HR control. The outcomes of these exercise capability and QoL analyses could not be generalized to all patients with permanent AF because all these study patients were in NYHA functional class I or II and patients with severe heart failure were not included.

## Conclusions

In patients with permanent AF, exercise capacity may not be affected by the stringency of rate control. Also, strict rate control may be associated with better QoL.

## Supplementary information

**Additional file 1: Table S1.** Multivariate Regression Analysis of Resting Heart Rate and PCS. **Table S2.** Multivariate Regression Analysis of Resting Heart Rate and MCS. **Table S3.** Multivariate Regression Analysis of Exercise Heart Rate and PCS. **Table S4.** Multivariate Regression Analysis of Exercise Heart Rate and MCS. **Table S5.** Pearson correlation between heart rate and SF-36 score item.

## Data Availability

The datasets used and/or analyzed during the current study available from the corresponding author on reasonable request.

## Abbreviations

6MWT: 6-min walk test
AF: Atrial fibrillation
CPET: Cardiopulmonary exercise testing
HR: Heart rate
MCS: Mental component summary
PCS: Physical component summary
QoL: Quality of life
RACE II: Permanent atrial fibrillation II
