# Supplementary material for: Influence of heart rate control on exercise capacity and quality of life in patients with permanent atrial fibrillation
Source: BMC Cardiovasc Disord. 2019 Dec 21;19:308. doi: 10.1186/s12872-019-01293-3 (PMC6925461; doi:10.1186/s12872-019-01293-3)
Supplement: Supplementary file 1 — Additional file 1: Table S1. Multivariate Regression Analysis of Resting Heart Rate and PCS. Table S2. Multivariate Regression Analysis of Resting Heart Rate and MCS. Table S3. Multivariate Regression Analysis of Exercise Heart Rate and PCS. Table S4. Multivariate Regression Analysis of Exercise Heart Rate and MCS. Table S5. Pearson correlation between heart rate and SF-36 score item. [file 12872_2019_1293_MOESM1_ESM.docx]

**Table S1.** Multivariate Regression Analysis of Resting Heart Rate and PCS

|  | β | Δβ | P |
| --- | --- | --- | --- |
| Gender | -34.788 | -0.306 | 0.186 |
| Age | -3.018 | -0.450 | 0.068 |
| Duration of AF | 3.588 | 0.570 | 0.021 |
| Hypertension | 3.017 | 0.028 | 0.897 |
| Diabetes | -36.642 | -0.281 | 0.162 |
| Hyperlipidemia | -26.923 | -0.237 | 0.366 |
| Valvular Disease | -18.493 | -0.106 | 0.648 |
| CHD | 21.470 | 0.140 | 0.499 |
| Smoke | 11.501 | 0.093 | 0.647 |
| BMI | -0.324 | -0.019 | 0.924 |
| SBP | 0-.293 | -0.094 | 0.653 |
| Resting HR | -1.218 | -0.470 | 0.066 |

β, Coefficients;Δβ, Standardized Coefficient; AF, atrial fibrillation; CHD, coronary heart disease; BMI, body mass index; SBP, systolic blood pressure; Resting HR, resting heart rate

**Table S2.** Multivariate Regression Analysis of Resting Heart Rate and MCS

|  | β | Δβ | P |
| --- | --- | --- | --- |
| Gender | -28.284 | -0.243 | 0.310 |
| Age | -2.842 | -0.414 | 0.105 |
| Duration of AF | 3.357 | 0.521 | 0.041 |
| Hypertension | 0.660 | 0.006 | 0.979 |
| Diabetes | -35.649 | -0.267 | 0.202 |
| Hyperlipidemia | -28.503 | -0.245 | 0.372 |
| Valvular Disease | -35.582 | -0.200 | 0.415 |
| CHD | 20.383 | 0.130 | 0.548 |
| Smoke | 2.264 | 0.018 | 0.933 |
| BMI | 0.040 | 0.002 | 0.991 |
| SBP | -0.400 | -0.125 | 0.567 |
| Resting HR | -1.271 | -0.479 | 0.072 |

β, Coefficients;Δβ, Standardized Coefficient; AF, atrial fibrillation; CHD, coronary heart disease; BMI, body mass index; SBP, systolic blood pressure; Resting HR, resting heart rate

**Table S3.** Multivariate Regression Analysis of Exercise Heart Rate and PCS

|  | β | Δβ | P |
| --- | --- | --- | --- |
| Gender | -16.956 | -0.149 | 0.515 |
| Age | -2.609 | -0.389 | 0.069 |
| Duration of AF | 5.018 | 0.798 | 0.002 |
| Hypertension | 20.277 | 0.187 | 0.336 |
| Diabetes | -36.527 | -0.280 | 0.135 |
| Hyperlipidemia | -37.442 | -0.329 | 0.195 |
| Valvular Disease | -57.659 | -0.332 | 0.136 |
| CHD | 31.474 | 0.205 | 0.298 |
| Smoke | 22.856 | 0.185 | 0.351 |
| BMI | -2.343 | -0.137 | 0.448 |
| SBP | -0.785 | -0.251 | 0.244 |
| Exercise HR | -0.705 | -0.487 | 0.020 |

β, Coefficients;Δβ, Standardized Coefficient; AF, atrial fibrillation; CHD, coronary heart disease; BMI, body mass index; SBP, systolic blood pressure; Exercise HR, exercise heart rate

**Table S4.** Multivariate Regression Analysis of Exercise Heart Rate and MCS

|  | β | Δβ | P |
| --- | --- | --- | --- |
| Gender | -7.293 | -0.063 | 0.786 |
| Age | -2.511 | -0.365 | 0.090 |
| Duration of AF | 4.939 | 0.767 | 0.003 |
| Hypertension | 18.892 | 0.170 | 0.386 |
| Diabetes | -36.721 | -0.275 | 0.147 |
| Hyperlipidemia | -40.738 | -0.350 | 0.175 |
| Valvular Disease | -78.150 | -0.439 | 0.057 |
| CHD | 31.490 | 0.200 | 0.315 |
| Smoke | 15.433 | 0.122 | 0.540 |
| BMI | -2.068 | -0.118 | 0.518 |
| SBP | -0.976 | -0.305 | 0.166 |
| Exercise HR | -0.801 | -0.540 | 0.012 |

β, Coefficients;Δβ, Standardized Coefficient; AF, atrial fibrillation; CHD, coronary heart disease; BMI, body mass index; SBP, systolic blood pressure; Exercise HR, exercise heart rate

**Table S5.** Pearson correlation between heart rate and SF-36 score item

|  | Exercise HR | | Resting HR | |
| --- | --- | --- | --- | --- |
| SF-36 score item | r | P value | r | P value |
| PF | -0.14 | 0.2493 | -0.08 | 0.5109 |
| RP | -0.68 | <.0001 | -0.37 | 0.0025 |
| BP | 0.01 | 0.9599 | -0.10 | 0.4451 |
| GH | -0.87 | <.0001 | -0.51 | <.0001 |
| VT | -0.85 | <.0001 | -0.47 | <.0001 |
| SF | -0.85 | <.0001 | -0.45 | 0.0002 |
| RE | -0.58 | <.0001 | -0.15 | 0.2252 |
| MH | -0.50 | <.0001 | -0.26 | 0.0347 |

PF, Physical functioning; RP, Role limitation due to physical problems; BP, Bodily pain, GH, General health; VT, Vitality; SF, Social functioning; RE, Role limitation due to emotional problems; MH, Mental health
